# Improving heart health among Black/African American women using civic engagement: a pilot study

**DOI:** 10.1186/s12889-016-3964-2

**Published:** 2017-01-24

**Authors:** Alison G. M. Brown, Linda B. Hudson, Kenneth Chui, Nesly Metayer, Namibia Lebron-Torres, Rebecca A. Seguin, Sara C. Folta

**Affiliations:** 1 Department of Public Health and Community Medicine, Tufts University School of Medicine, Boston, MA USA; 2 Moakley Center for Public Management, Suffolk University, Boston, MA USA; 3 Tufts University Friedman School of Nutrition Science and Policy, Boston, MA USA; 4 Cornell University College of Human Ecology, Ithaca, NY USA

**Keywords:** Cardiovascular disease, Blacks/African Americans, Lifestyle behaviors Socioecological model, Health disparities

## Abstract

**Background:**

Despite increased risk for cardiovascular disease (CVD) and related conditions, evaluations of health interventions indicate that Black/African American women are less likely to benefit than their white counterparts and are not as likely to engage in behaviors that reduce CVD risk. The purpose of this study was to test the feasibility and effectiveness of civic engagement as an intervention strategy to address heart health in Black/African American women.

**Methods:**

Using a quasi-experimental pre-post study design, civic engagement was tested by convening a convenience sample of self-identified Black/African American women, ages 30–70 years, English-speaking, and BMI ≥25.0 (*n* = 28) into “Change Clubs” in four churches. Feasibility was examined through adherence, satisfaction, retention, and ability of Change Clubs to meet at least 50% of self-identified action steps for community change. Effectiveness data included: dietary intake, measures of physical activity, cardiorespiratory fitness, blood pressure, and anthropometrics. Psychosocial factors hypothesized to serve as the mechanisms by which civic engagement enacts behavior change were also assessed.

**Results:**

At baseline, the study sample (*n* = 28) had a mean age of 50.5 y; 53.6% had an associate degree or higher; 60.7% had an income of $35,000 or higher; and 57.4% were employed full time. At the conclusion of the study, all participants were satisfied with the progress of their Change Club and with the overall experience and Change Clubs met their self-identified action steps for community change. The intervention had a significant effect on finish time on the cardiorespiratory fitness test (*p* < 0.001) and systolic blood pressure (*p* < 0.001).

**Conclusions:**

Study results suggest feasibility and evidence of preliminary effectiveness of using a civic engagement approach to address behavior change in a way that is appealing and acceptable to Black/African American women.

**Trial registration:**

NCT02173366

## Background

The prevalence of diet- and behavior-related diseases and conditions, such as overweight and obesity, hypertension, and cardiovascular disease (CVD), are higher among Black/African Americans in comparison to other racial groups [1]. For example, 82% of Black/African American women age 20 years or older are overweight or obese, compared to 61% of white women, and few Black/African American women meet dietary or physical activity guidelines [1]. According to the American Heart Association, the prevalence of hypertension and CVD among Black/African American females (≥20 years) is 46% and 48%, respectively [1].

Despite increased risk for CVD and related conditions, Black/African American women are not as likely to engage in behaviors that reduce CVD risk [1], and evaluations of health interventions indicate that Black/African American women are less likely to benefit than their white counterparts [2–5]. These differences in effectiveness could be due to a variety of factors including, cultural appropriateness of interventions, time constraints and adherence to interventions, and resources [2–5]. The relatively few interventions developed specifically for Blacks/African Americans indicate that it is possible to achieve significant improvements in risk behaviors and biological parameters when culture, values, beliefs, and unique barriers are taken into account [6–14]. Despite this evidence and the large disparities in CVD risk, few gender- and culture-specific CVD prevention interventions exist [8, 11–15].

The African American Collaborative Obesity Research Network (AACORN)—a collaboration of U.S. researchers, scholars, and community-based research partners dedicated to addressing weight-related concerns in Black/African American communities—emphasizes the importance of engaging community members in the planning and implementation of interventions [16]. Specifically, the AACORN research paradigm recommends an eco-social, community-engaged approach to behavior change as most appropriate and effective in Black/African American communities [16]. AACORN further asserts that recognizing the historical and social contexts, cultural and psychosocial processes, and the physical and economic environments that influence health behaviors among the Black/African American population is essential to effective health promotion in this population. Interventions should therefore not only embody the culture of Black/African American communities (i.e., values of interconnectedness, religiosity, and importance of family), but also be framed in the context of environmental and policy-level factors [17]. An additional critical consideration noted by AACORN is the heterogeneity (i.e., ethnicity, socioeconomic status, and education) among the Black/African American population and how an understanding of and responsiveness to this multidimensionality of the study population must underlie the development of programs and interventions if they are to be effective [4].

The objective of this pilot study is to test the operationalization of the AACORN paradigm through a civic engagement approach. Civic engagement, defined as “individual and collective actions designed to identify and address issues of public concern” [18], is an innovative approach to behavior change. In 2011, several members of the research team conducted a preliminary project examining this strategy for health promotion among predominantly white women [19]. Participants gathered in “Change Clubs” (also referred to as the civic engagement approach), and engaged in a community audit designed to increase awareness about their food and physical activity environments. They then self-identified a specific community change objective, such as improving snacks in after school programs or advocating for traffic calming measures to improve community walkability. Through the Change Club curriculum, groups were led through a process of setting action steps and monitoring progress. Seven of eight clubs were successful in meeting at least half of their benchmarks [19]. Although not evaluated in that project, this process may also lead to change in individual health behavior.

**Fig. 1:**
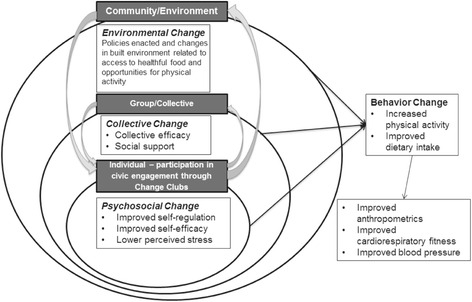
Study theoretical framework

Informed by the AACORN paradigm, our study framework (Fig. 1) depicts the mechanisms by which civic engagement may affect behavior change at each level of the eco-social model (i.e., individual, interpersonal, organizational, community, public policy) [20]. At the individual level, civic engagement fosters self-regulation (goal-setting and monitoring) and self-efficacy, which are associated with healthier dietary behaviors, regular physical activity, and weight status [21–27]. The study framework also suggests civic engagement will decrease perceived stress through empowerment, or acting in a self-determined manner to effect change in the public sphere [28]. At the group/collective level, by identifying and changing environmental factors affecting community health, the group gains a sense of collective efficacy to create collective change [29]. The group itself provides social support, known in the literature to positively affect health behaviors [30, 31]. It is also anticipated that there is a positive interaction between individual-level changes and collective efficacy of the group by which changes at each level reinforce the other [29]. At the community/environment level, civic engagement is designed to promote environmental and policy changes that further support personal and collective efficacy and behavior change through reciprocal determinism [29]. Per the AACORN paradigm, the civic engagement process reveals community strengths that can be leveraged for health promotion [16].

It is hypothesized that the Change Club civic engagement approach would be culturally appropriate for Black/African American women, since it places the focus on collective health and builds on cultural strengths such as interconnectedness, importance of community, and care for others. The purpose of this study was to determine feasibility and preliminary effectiveness of civic engagement as a way to affect changes in CVD-related behaviors and outcomes in this population. The study also aims to determine trends and obtain data to allow calculation of an adequately powered sample in a larger study.

## Methods

### Design, sample, and setting

A quasi-experimental pre-post study design was used and the Tufts University Health Sciences Institutional Review Board approved all study procedures.

**Fig. 2:**
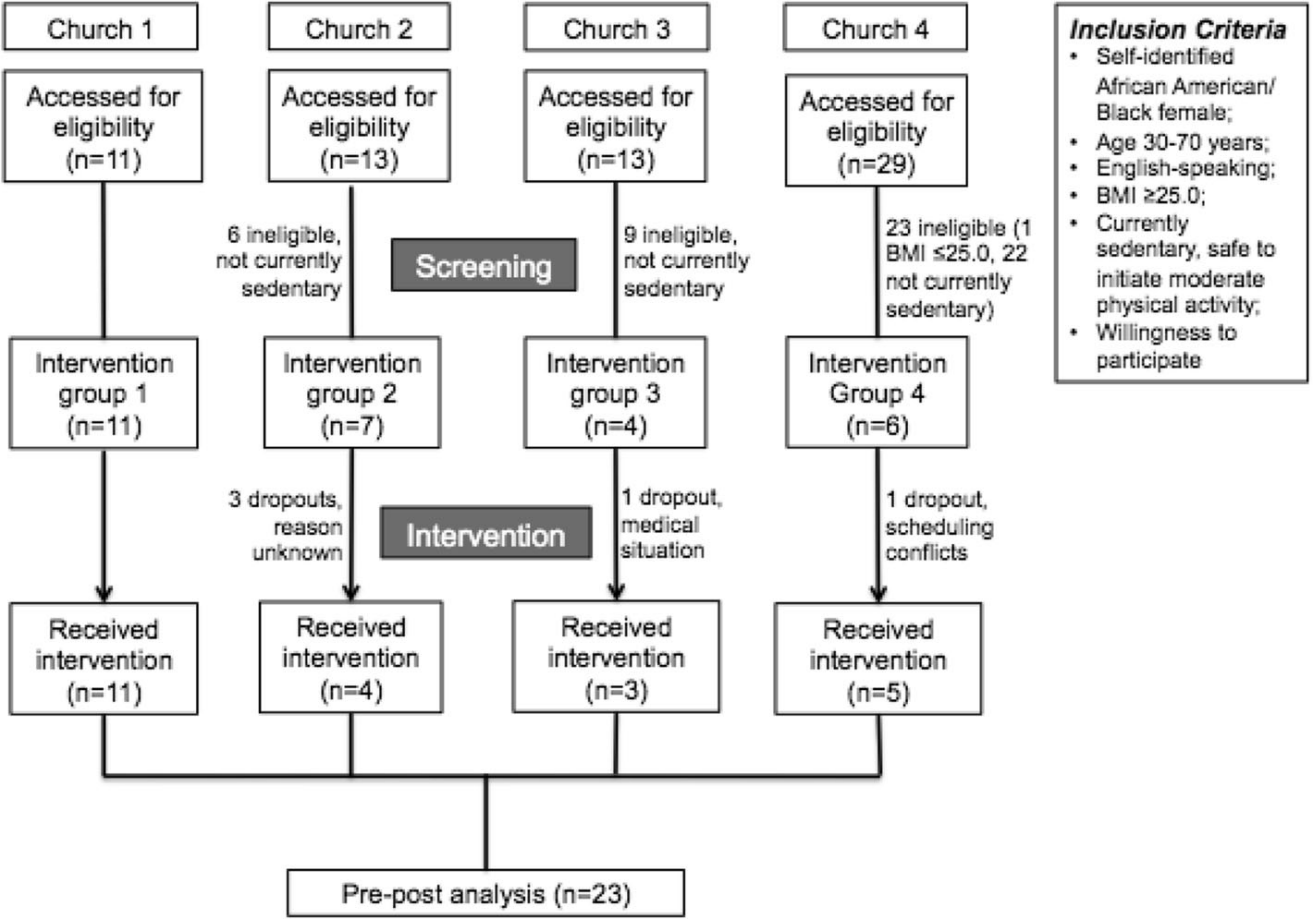
Study consort map

Participants were recruited in collaboration with four churches located in predominantly Black/African American neighborhoods in Boston, Massachusetts, United States. Churches were selected based on geographic location and existing connections with these faith-based institutions. Informational sessions in the format of presentations about the study were conducted at each of the churches and promotional flyers were posted onsite. The research coordinator screened potential participants who expressed initial interest in the research study by telephone. Inclusion criteria were self-identifying as Black/African American; female; age 30–70 years; English-speaking; BMI ≥25; currently sedentary (not meeting Physical Activity Guidelines for Americans; and safe to initiate moderate physical activity based on the PARQ) [32]. Exclusion criteria included failure to provide informed consent; for those who had any contraindications to physical activity per the PAR-Q, failure to obtain physician consent to participate; participation in any other lifestyle modification program; current use of either prescription or over-the-counter weight loss medications; inability to communicate due to severe, uncorrectable hearing loss or speech disorder; severe visual impairment (if it precludes completion of assessments and/or intervention); planning to move outside of area within 6 months; and self-reported pregnancy, since weight loss is inadvisable [32]. Women who were eligible based on their responses to the telephone screening, were invited to participate in the study. Prior to the baseline assessments, each participant reviewed and signed the informed consent agreeing to participate in the study. Figure 2 depicts the study flow and sample sizes.

### Intervention

The 6-month intervention was developed from elements of the Change Club and the StrongWomen – Healthy Hearts curriculum, a community-based CVD prevention program for midlife and older women [19, 33] (See Table 1 for the Intervention Overview). The intervention herein will be referred to as the Change Club civic engagement curriculum. The Change Club and StrongWomen – Healthy Hearts curriculum were adapted for cultural appropriateness through the consideration of food recommendations, language choice, and layout and design of curriculum materials. Change Club members met weekly for 6 months and participated in a step-by step process using the civic engagement approach.

**Table 1:** Intervention overview

	Month	General Overview	Civic Engagement Topic Covered	Nutrition and Physical Activity Topic Covered
Planning Phase	1	Togetherness and Unity	Intervention introduction	Heart healthy food discussion
			Rapport building	Foods that contribute to and prevent heart disease
			Establish purpose, group name, and group norms	Discussions on added sugar, sodium, portion sizes, hunger cues, and using a pedometer
			Community walking tour and assessment	
	2	Determining the Community Need	Interview skills training	Importance of setting S.M.A.R.T. goals around nutrition and physical activity
			Identification of community leaders	
			Importance of setting S.M.A.R.T. goals for group project	
				Discussions on fruit and vegetables, heart healthy fats
			Identify community needs	
			Define purpose of group	
	3	Planning for Next Steps	Leadership skills within the group	Discussions on eating whole grains, low fat and non-fat dairy, heart-healthy proteins
			Identification of assets, skills, and strengths of group members	
			Develop action plans to execute purpose of the group	
			Task delegation and action items for each group member	
Implementation/Action Phase	4	Action, Part 1	Execution of action plan	Monitoring of nutrition and physical activity goals
			Review progress in meeting benchmarks and major group accomplishments	
	5	Action, Part 2		
	6	Next Steps	Complete action steps	
			Summarize group accomplishments	
			Establish a plan for next steps (if applicable)	

The first three months of the intervention (planning phase) were facilitated by research staff at which time the Change Clubs were guided through the process of planning, visioning, stakeholder identification, asset mapping, and goal setting (using action steps, or specific, measurable action items toward goal attainment). For example, Month 1 focused on building the rapport of the group, identity formation, and establishing group norms. Month 2 included identification and assessment of community needs through interviews with key community leaders and community walkabouts and selecting a central goal related to a nutrition or physical activity issue of concern in their community. In Month 3, the group created a plan of action to execute the self-identified group project, including task delegation and action items for each group member. During these three months, research team members either facilitated or co-facilitated the Change Clubs. The facilitators each had professional experience with group facilitation and the Change Club civic engagement curriculum. Additionally, at each session during this phase, participants received basic nutrition education from the StrongWomen – Healthy Hearts curriculum, designed to interweave with the civic engagement approach. For example, as groups participated in awareness activities focusing on food environments (i.e., accessibility and affordability of healthy foods and produce) during Month 2, they also examined and discussed their personal environments and circumstances (i.e., impact of work schedule and family dynamics on eating habits).

The final three months of the intervention (the implementation/action phase) was facilitated by a self-selected leader and included the period when the group implemented the identified project. The action steps and benchmarks established during the planning phase of the intervention guided the group’s activities. As a process evaluation measure, research staff monitored the fidelity to the curriculum through site visits during those last 3 months.

Each participant received a $150 gift card at each assessment (pre- and post-), each participating church (leadership and administration) received $200 for assistance in recruitment and provision of space, and the Change Clubs received $1,000 each to support their planned community activities.

### Measures

Participants completed a questionnaire at baseline to provide demographic information: age, marital status, occupational status, education level, income level, and household characteristics. Most questions were based on those used in national surveys, i.e., the U.S. Census and the Behavioral Risk Factor Surveillance System [34].

Feasibility of the Change Club study was measured by rates of retention and adherence, with retention defined as the percentage of those who completed the pre- post- assessments (6 months), and adherence operationalized as the number of sessions attended during the first 3 months of the study. To provide further insight into feasibility in terms of acceptability of the intervention and group progress toward stated goals, the post-assessment included acceptability and satisfaction questions. The identified Change Club leader and one other randomly selected participant from each group also participated in key informant interviews (KIIs) post-intervention. Interviews were conducted by two research team members and were guided by a semi-structured interview guide. Key topics included perceptions of roadblocks and factors leading to success; feedback on research team support; experiences with connecting and communicating with political decision makers and stakeholders and others in the community; perceptions about group dynamics and communication among members; and thoughts about the overall experience. Data analysis is described below.

Anthropometrics, psychosocial, physiological, and behavioral outcomes were collected within two weeks prior to the start of the Change Club intervention (pre) and again within two weeks following the conclusion of the 6-month intervention (post). Demographic variables were only collected during the pre-assessment. See Table 2 for a complete list of measures.

**Table 2:** Assessed measures

	Outcome	Method	Reliability and Validity
Psychosocial change (pre and post)	Self-regulation	Self-regulation (14-item)	Saelens, et al., 2000 [35]
	Perceived stress	Perceived Stress Scale (14-item)	Cohen, Kamarck, & Mermelstein, 1983 [37]
	Self-efficacy	Weight Efficacy Lifestyle Scale (20-item)	Clark, et al., 1991 [65]
		Exercise Self Efficacy Scale (5-item)	Marcus, Selby, Niaura, & Rossi, 1992 [39]
	Civic engagement	Civic Engagement Scale (14-item)	Doolittle & Faul, 2013 [41]
Collective change (pre and post)	Collective efficacy	Collective Efficacy scale (8-item)	Sampson, Raudenbush, & Earls, 1997 [36]
	Social support	Sallis Social Support Scales for Eating and Exercise Behavior (23-item)	Sallis, et al., 1987 [40]
Behavior change (pre and post)	Physical activity	Accelerometry GTX3	
		IPAQ, long form	Blair et al., 1991 [66]
	Fruit and vegetables	24-h recall	Johnson, Driscoll, & Goran, 1996 [44]
	Sodium		
Physiological (pre and post)	Body weight	Digital floor scale (3 measurements)	
	Height (pre)	Portable stadiometer (Seca 214)	Lohman, 1992 [67]
	Body fat percentage	Bioelectric impedance (Body Composition Analyzer)	Lopez, O’Connor, Ledoux, & Lee, 2011 [51]
	Waist circumference	Retractable tape measure	Lohman, 1992 [67]
	Cardiorespiratory fitness (VO_2_ max)	Rockport 1-mile walk test	D’Alonzo, Marbach, & Vincent, 2006; [68] Kline et al., 1987 [47]
	Blood pressure	Automated blood pressure monitor (2 measures within 5 mmHg)	Pickering et al., 2005 [69]
Demographic (Covariates) (pre)	Age	Participation information survey	BRFSS and study-developed questions
	Marital status	Participation information survey	
	Occupational status	Participation information survey	
	Education level	Participation information survey	
	Income level	Participation information survey	
	Household characteristics	Participation information survey	
	Acculturation	African American Acculturation Scale (47-item)	Klonoff & Landrine, 2000 [70]
Feasibility	Retention	Post self-reported survey	
	Adherence (# of sessions attended)	Post self-reported survey	
	Acceptability	Post self-reported survey	
		Key informant interview	

The assessed psychosocial factors hypothesized to serve as the mechanisms by which civic engagement enacts behavior change per our study framework included: self-regulation, collective efficacy, perceived stress (proposed to be reduced through empowerment), self-efficacy, and perceived social support [35–40]. The Civic Engagement Scale was used to measure civic engagement [41]. Because of the hypothesized cultural relevance of the civic engagement approach, data were collected from the African American Acculturation Scale, which measures dimensions of African American culture, whereby a higher score means greater acculturation to the African American culture [42, 43].

Dietary intake was based on two telephone-based, 24-h dietary recalls conducted by trained research personnel, either a registered dietitian or graduate students in nutrition, using the multiple-pass protocol [44]. The multiple-pass method is a multi-step process designed to enhance complete and accurate food recall and reduce respondent burden [44]. Telephone recalls were unannounced. Data were entered using the Nutrition Coordinating Center at the University of Minnesota Food’s Nutrition Data System for Research (NDSR) [45].

Physical activity was measured both objectively by accelerometry (ActiGraph Model GT3X), worn for seven consecutive days, and by self-report over the previous 7 days (International Physical Activity Questionnaire, long form) [46]. Cardiovascular fitness was measured using the Rockport 1-mile walk test, which has been validated with racially diverse sedentary women [47]. Blood pressure was measured to the nearest 1 mmHg using a validated American Heart Association protocol [48, 49].

Body weight was measured using a digital floor scale (Seca 876). Height was measured, using a portable stadiometer (Seca 214) according to the procedures of Lohman [50]. Waist circumference was measured using a research-grade retractable tape measure (Seca 200), also using Lohman procedures [50]. Percent body fat was measured by bioelectric impedance (Body Composition Analyzer MC – TBF-410, Tanita Corp., Tokyo, Japan) and estimated using an equation developed and validated for Black/African American women [51].

### Statistical analysis

Descriptive statistics of the demographic information were composed and tabulated. Potential confounding variables were selected from the following: age (year), education (≤high school or > high school), annual income (≤US$35,000 or > US$35,000), partnered vs. not partnered, employed full-time vs. part-time or not employed, nativity (born in US or not), and the African American Acculturation Scale score. Selections were done through fitting each of these potential confounding variables into the model with the pre-post indicator as independent variables. Observations were made on how much the estimate of the pre-post indicator changed. Variables were categorized as a potential confounder if its inclusion caused the regression estimate of the pre-post indicator to change more extreme than 10%.

Two regression models were performed for each outcome, an unadjusted model using only the pre-post indicator as the predictor; and an adjusted model that controls for confounding variables identified by the method described above. We opted for using mixed models with individual identification numbers as the random intercept so that each participant would have her own intercept. We favored the mixed model approach over paired sample *t*-test because mixed model can tolerate some degree of imbalanced data, which was introduced by five participants who were lost to follow up. Due to the low sample size, adjustments could not be performed for the site (church) as a random effect within which the individuals were nested as the models failed to converge. Control for churches’ effect was carried out as a fixed independent variable. Stata Statistical Software: Release 14 was used for the data management and analysis [52]. While the *a priori* type I error rate was set at 5%, this is a pilot study aiming to understand feasibility and estimate effect size, therefore, the *p*-values of this analysis should be evaluated with caution.

### Qualitative data analysis

The research assistants digitally recorded and transcribed the KIIs. Data were analyzed using the qualitative analysis technique, inductive thematic analysis, in which meaningful patterns and themes were identified and examined [53]. First, the team developed the initial codebook based on emergent themes from the KII transcripts. The codebook was refined based on coding several initial transcripts. Inter-coder reliability was established at 80% agreement or greater; in the few instances where this was not achieved, researchers met to discuss discrepancies and further refined the codebook to clarify code definitions. The process was repeated until inter-coder reliability was established. NVivo 10 software was utilized to assist in the coding and analysis the process (QSR International, Australia) [54].

## Results

### Baseline

Baseline data were obtained for 28 women. Demographic characteristics of the sample are summarized in Table 3. The sample had a mean age of 50.5 years; 53.6% had obtained an associate degree or above; 60.7% had an income of $35,000 or above; and 57.4% were employed full time. As shown in Table 4, at baseline across the four churches (*n* = 28) the mean weight was 204.8 lbs (95% CI 190.4, 219.3, and mean body fat percentage was 45.6% (95% CI 43.5, 47.8). Mean systolic blood pressure was 135.4 mmHg (95% CI 127.0, 143.7), and mean diastolic blood pressure was 81.2 mmHg (95% CI 77.5, 84.9). Participants self-reported 3325 MET-minutes per week of physical activity and at baseline, the average time for the walking test was 22 min and 6 s (95% CI 20.7, 23.4). Participants consumed an average of 1834 kcals/day (95% CI 1289, 1952), 2790 mg sodium/day (95% CI 2239, 3342), 1.3-cup equivalents of fruit/day (95% CI 0.8, 1.9) and 2.2-cup equivalents of vegetables/day (95% CI 1.6, 2.7).

**Table 3:** Demographic characteristics of study participants at baseline (*N* = 28)

	Mean (SD)
Age, year	50.5 (9.4)
	N (%)
Education	
High school graduate/GED certificate or less	13 (46.4)
Associated degree or some college	3 (10.7)
College degree	6 (21.4)
Master’s degree/graduate degree	6 (21.4)
Income	
Less than $24,999	7 (25.9)
$25,000–49,000	7 (25.9)
$50,000–74,999	5 (18.5)
Above $75,000	8 (29.6)
Living with a partner	
No	16 (57.1)
Yes	12 (42.9)
Employment status	
Not employed	5 (17.9)
Part time	7 (25.0)
Full time	16 (57.1)
Born in the U.S.	
No	6 (21.4)
Yes	22 (78.6)

*SD* Standard deviation

Frequencies may not add up to 28 due to missing data

**Table 4:** Study outcomes

	Pre-Intervention, Mean (SD)	Post-Intervention, Mean (SD)	Unadjusted coefficient (95% CI)	Adjusted coefficient (95% CI)
Anthropometrics				
Weight, lb	204.8 (35.7)	206.7 (39.2)	0.513 (−2.112, 3.137)	0.544 (−2.069, 3.156)^b^
Waist circumference, cm	102.9 (12.9)	108.5 (16.2)	4.770** (1.555, 7.986)	- ^a^
Adjusted body fat, %	44.0 (4.8)	42.7 (5.0)	−0.644 (−1.613, 0.324)	- ^a^
Dietary intake				
Dietary energy intake, kcal/day	1834 (830)	1544 (416)	−249.1† (−542.3, 44.14)	−233.9 (−533.7, 65.85)^d^
Fruit intake, serving/day	1.3 (1.5)	1.5 (1.6)	.798† (−0.019, 1.616)	0.761 (−0.119, 1.641)^c^
Vegetable intake, serving/day	2.2 (1.4)	2.3 (1.9)	0.424 (−0.624, 1.472)	0.505 (−0.542, 1.553)^f^
Sodium intake, mg/day	2790 (1395)	2511 (706)	−256.0 (−855.9, 344.0)	−307.3 (−938.9, 324.2)^g^
Physical activity				
MVPA time, min/day	13.0 (8.8)	12.6 (9.2)	0.882 (−2.331, 4.096)	0.700 (−2.464, 3.865)^e^
Total accelerometry count, 1000 counts/day	210.0 (74.3)	214.6 (94.6)	13.02 (−23.64, 49.67)	9.230 (−26.82, 45.28)^b^
IPAQ, MET.min.wk^-a^	3237 (3490)	4738 (4206)	1727* (379.98, 3416)	1619† (−62.49, 3302)^b^
Cardiovascular fitness				
Time to finish VO2max test, min	22.1 (3.4)	20.2 (2.5)	−1.869*** (−2.892, −0.845)	- ^a^
Blood pressure				
Systolic blood pressure, mmHg	137.4 (23.8)	122.5 (11.9)	−12.73*** (−18.94, −6.512)	- ^a^
Diastolic blood pressure, mmHg	81.9 (10.5)	77.3 (9.6)	−3.312* (−6.407, −0.217)	−2.83† (−5.901, 0.244)^c^

All models adjusted for unique church ID number as fixed effects and personal ID number as random intercept

†: *p* < 0.10; *: *p* < 0.05; **: *p* < 0.01; ***: *p* < 0.001

Pre-post differences may not be equal to the unadjusted coefficient due to slight imbalance in responses caused by missing data

^a^No confounding variables identified

^b^Adjusted for place of birth

^c^Adjusted for place of birth and marital/partner status

^d^ Adjusted for age

^e^Adjusted for place of birth, age, marital/partner status, education level, and employment status

^f^Adjusted for place of birth, marital/partner status, and score on African American Acculturation Scale (AAAS)

^g^ Adjusted for age and score on African American Acculturation Scale (AAAS)

### Feasibility

All participants were satisfied with the intervention: 100% responded they were “very satisfied” or “generally satisfied” with the overall experience; and 100% responded they were “very satisfied” or “generally satisfied” with the progress their Change Club had made with respect to meeting self-identified action steps and achieving their overall goal. All four Change Clubs met all of their self-identified action steps for community change within approximately 6 months. Study retention was 82.1% and adherence was 79.0% supporting the feasibility of the intervention. The Change Club initiatives included provision of organizing monthly cooking demonstrations for the community, education and skills for healthier eating among food pantry patrons, compiling a cookbook and healthy lifestyle tips to support parents in providing healthy foods for their children, and organizing a health fair (See Table 5).

**Table 5:** Group purpose, example action steps, and outcome of each change club

Group Purpose	Example Action Steps	Outcome
“*To increase access to heart-healthy food among those most in need”*	Decide upon heart healthy recipe for each cooking demonstration	Conducted a monthly heart-healthy cooking demonstration for the community
“*To increase access to and use of healthy and culturally appropriate foods in the community”*	Decide on an appetizer and main dish to prepare during the taste testing	Implemented taste testing and nutrition education to food pantry patrons of the church
“*To provide information to the greater church community to promote healthier lifestyle”*	Collect permission slips from parents of children at the church-affiliated school to use child’s pictures in cookbook	Developed a heart healthy cookbook for the parents of the school affiliated with the church and healthy lifestyle messages to include in the church's bulletin
“*To inform and educate the community in making healthy eating and fitness choices* “	Identify and contact vendors across multiple disciplines (e.g., law enforcement, food industry, health care and emergency services, physical fitness, land conservation)	Implemented a holistic wellness fair designed to serve the geographic catchment area of the church

Based on the qualitative analysis of the KIIs, emergent themes included positive perceptions of the overall experience, appreciation of connecting with others in the group, strengths and skills of the group members, the barrier of time, and the challenge of managing different viewpoints. See Table 6 for major themes and supporting quotes. Participants and leaders felt their Change Clubs were successful and attributed this success to effective teamwork and a diversity of skills within the groups.. Study participants indicated overall satisfaction with the support from the research team and expressed gratitude for the opportunity for empowerment and cultivation of leadership skills. In terms of the civic engagement aspects of the intervention, emergent themes focused on positive aspects, such as connecting with others in the group and being motivated by them. Meanwhile, time was the biggest challenge to participation. Key themes for group dynamics included: acknowledgement and appreciation of the strengths that individual Change Club members brought to the group, view that the group was greater than the sum of its parts, establishment of positive social connections, challenge of managing different views, and concern about uneven participation by group members.

**Table 6:** Key informant interview results

Theme	Supporting quotes
*Overall Experience* • The overall experience was positive	“*For the congregation, the people we spoke to, the people I spoke to, are very enthusiastic with what we shared with them and that we are going to continue. We were recognized as a group that had a successful six months”* *“Knowledge. Not so much about the health. I think my eating habits have probably worsened. But it made me care about my health. I use my pedometer and I want to get fit. So those are the things. I learned patience. I am retired so it was interesting working on a project like this and using all the skills that one acquires I guess.”*
*Civic Engagement Piece* • Many participants noted positive aspects related to connecting with others in the group and being motivated by them • Working within a familiar neighborhood and community (that of the church) facilitated the group project • Time was the biggest challenge to participation	*“It was wonderful we worked together we were cooperative and everyone had assignments and there was a leader basically but everyone had a strength in something was how it worked for our group.”* *“We met on Sundays and sometimes that time became a crunch time because some of the members had other committee meetings to attend other members lived in other places.”* *“Quite a few people in our Change Club, at least two, are native Bostonians. Because they’re native Bostonians then they know the area, they know people. I think out of the group all of us are familiar with different segments, and I think all of us are … voters, so I think all of us kind of knew where we could go.”*
*Group Dynamics* • Strengths that individual Change Club members brought to the group were acknowledged and appreciated • The group was at times viewed as greater than the sum of its parts • Positive social connections were made • It was sometimes challenging to manage differing views • There was concern about uneven participation by group members	*“The team itself we had a good coach and the individual people who participated everyone was willing to share. And learn from each other and everyone had to contribute everyone made a contribution.”* *“We had one person who was like a walking encyclopedia—we don’t have encyclopedias anymore, but—she was full of resources and I knew if we needed to know anything, she would know.”* *“Everybody knew that any idea was welcome on the floor and sometimes we ran up on a solution that would not have come about if it weren’t for the freedom to brainstorm, so I definitely think people communicated very well.”* *“In the end I cared and I didn’t expect to care so much. You know ‘I’ll never see these women again. Our lives are very different.’ And just move on, but it didn’t turn out that way.”* *“When people were pushed against the wall, or when questions were asked, I mean people just shut down. Trying to engage everybody and bring them back in when someone shut down because they didn’t agree with what was going or they felt uncomfortable.”* *“I think we had people who could be insensible. People who weren’t really committed. At times I felt I was overbearing and over-dominating and very focused on trying to get the task completed. There was gridlock sometimes, we had reach an impasse and I had to call [our original facilitator] to intervene and run some things past her.”*

### Psychosocial factors

All of the psychosocial variables (self-regulation, collective efficacy, self-efficacy, perceived social support), except perceived stress changed in the expected direction [40]. However, none achieved statistical significance (data not shown).

### Outcome measure

Pre-post changes in dietary outcomes were in the expected direction, although none achieved statistical significance. Total daily caloric intake decreased (−233.9 calories, 95% CI −533.7, 65.8); mean daily intake of fruits (0.76 cups/day, 95% CI −0.12, 1.64) and vegetables (0.50 cups/day, 95% CI −0.52, 1.55) increased; and mean daily intake of sodium decreased (−307.3 mg/day, 95% CI −938.9, 324.2).

For physical activity, participants self-reported 3325 MET-minutes per week at baseline. Both the adjusted and unadjusted estimates indicate significant pre-post increases in physical activity (adjusted model: 1619, 95% CI 40, 3416). Accelerometry data, however, did not show significant changes although these outcomes (moderate or vigorous physical activity time and total accelerometry count) were in the expected direction (9.23 1000 counts/day, 95% CI −26.82, 45.28).

The intervention had a significant effect on the finish time of the 1-mile walk test (−1.89 min, 95% CI −2.89, −0.84, *p* < 0.001). The intention was to use the walk time and final heart rate to calculate an estimated maximal volume of oxygen uptake (VO_2max_) with a validated regression equation, however, the average walk times were too long for the estimation formula to be applicable [47]. Walk times are only reported. Heart rates were within the targeted range for this age group, suggesting that participants were exerting themselves adequately.

There were also significant pre-post changes in systolic blood pressure (−12.73 mmHg, 95% CI −18.94, −6,51, *p* < 0.001).

As shown in Table 4, although not significant, mean body weight increased (0.54, 95% CI −2.07, 3.16), with a pre-post range of 1.9 lbs for body weight. Mean waist circumference increased significantly (4.77, 95% CI 1.56, 7.98, *p* < 0.05) with a pre-post range of 5.6 cm. Body fat percentage, while not significant, changed in the expected direction (−0.64, 95% CI −1.61, 0.32).

## Discussion

The findings suggest that a civic engagement approach to behavior change among Black/African American women demonstrates a level of feasibility and preliminary effectiveness. There are notable strengths and weaknesses of the study. One strength is the demonstration of feasibility via several indicators. Observed rates of adherence and retention were comparable and in some cases better than in previous weight loss interventions that only included Black/African American women. For example, in a review by Fitzgibbon et al., retention rates ranged from 72% to 80%, and adherence was 83% in the one quasi-experimental study that included this metric [2]. In comparison, retention and adherence rates among Black/African American women subjects in interventions including multiple races were as low as 37% and 41%, respectively [2]. Our study therefore further supports the literature suggesting that interventions may be most acceptable to Black/African American women if they are culturally appropriate and tailored. The results should be interpreted with caution, however, given that adherence was not measured for the last 3 months of the intervention (discussed more below). Additionally, the somewhat higher financial inventive designed to compensate for the participant burden may have influenced the response rate, and the feasibility of sustaining this incentive amount in future large studies should be re-examined. Post-intervention results from both the quantitative and qualitative sources indicate that the study participants were highly satisfied with their Change Club experience. For example, a key theme of the KIIs was that the overall experience was described as being positive. The KIIs suggested that the participants believed they were successful in inspiring and motivating their communities suggesting a positive influence of the empowerment and civic engagement. Study results also indicate preliminary effectiveness, with pre-post improvements in dietary intake, physical activity levels, cardiovascular fitness, and blood pressure. Considering the prevalence of hypertension among Black/African American women, the significant decrease in systolic blood pressure is promising. While changes in blood pressure are often seen with weight loss, interestingly these changes were present independent of changes in weight. Physical activity is considered an independent contributor to lowering blood pressure, and may have contributed to the reductions in systolic blood pressure among study participants [55–57].

Despite the promising results, there are limitations that should be discussed. It was not feasible to obtain attendance data for the final 3 months of the intervention after the research facilitator stepped away. Additionally, while the intervention was originally designed for the Change Clubs to advocate for policy changes within their communities, a study finding worthy of further investigation is the domain of influence and impact chosen by the Change Clubs. Each Change Club identified a community-level, programmatic intervention to implement over the 6-month study instead of reaching the (arguably) more impactful policy-level domain. This finding could be attributed to a greater confidence within the Black/African American community of grassroots community processes and neighborhood-based programs rather than political advocacy and engagement with historically disenfranchising political structures [58, 59]. For example, none chose to try to enact broader change by engaging in political advocacy. The curriculum may need to be enhanced to bolster the aspect of political engagement and activism. The Change Clubs, however, decided on manageable programs to implement within the timeframe, and realized success at meeting their action steps. This is supportive of the study framework, in that setting and achieving realistic goals may have increased self-regulation and self- and collective-efficacy, leading to behavior change. Although not adequately powered to see changes in the psychosocial outcomes, pre-post changes in these factors are consistent with the study framework. Anticipated future work will include a larger, randomized, controlled trial adequately powered for mediation analysis to best evaluate the hypothesized mechanisms.

Another noteworthy unanticipated outcome included the increase in perceived stress post intervention, although the change was not statistically significant. This was unexpected given the anticipated increase in empowerment, as well as the increase in social support and camaraderie through group interactions. A possible explanation for this finding is that stress increased due to the additional obligation of being a part of the Change Club. This explanation is supported by the qualitative data, in which the participants described time constraints, uneven participation by group members, and conflicting commitments. Creative strategies to overcome barriers and the stress of participation should therefore be explored in future studies. These might include the facilitation of off-site participation through conference calls and video conferencing.

There is a growing body of evidence on culturally adapted interventions specifically designed to improve diet and weight outcomes for Black/African American women. In a systematic review of interventions with culturally adapted strategies for this at-risk target population, the authors categorized culturally adapted strategies as peripheral (targeted recruitment of Black/African Americans in the media and community), constituent–involving (engagement of the community in the planning and implementation of the intervention), socio-cultural (address themes such as spirituality, religiosity, faith, traditional and cultural foods, social support, barriers, and body image), and evidential and linguistic (consideration of scientific evidence and participant literacy levels) [60]. The review found more significant changes in behavior and weight when constituent-involving strategies were used, which is the strategy utilized in the Change Club civic engagement curriculum.

A finding also noted by Kong et al. and in the AACORN paradigm is the need to consider ethnic diversity among the Black/African American population and further examination of how this will influence the development of future interventions [16, 60]. In this study, recruitment of foreign-born women was somewhat reflective of the state’s Black/African American population (21.4% foreign-born in the study vs. 32.8% in the state) [61]. Although data are not shown, score on the African American Acculturation Scale was not found to be associated with place of birth. However, other results from this study suggest the importance of place of birth in predicting changes in weight, body fat percentage, diastolic blood pressure, and total accelerometry counts. As Kong et al. described, involving the community in the development and implementation of interventions is particularly critical to better understand the target population and recognize the heterogeneity among the group [60]. Future studies with larger sample size should pay greater attention to how cultural variables and ethnicity relate to study outcomes and possible mediation of the influence of the intervention.

The intent of this pilot study was to determine trends and obtain data that would allow for calculation of an adequately powered sample in a larger study, should results prove promising. By design, the sample size was small with a goal of recruiting 40 participants from four churches. Due to unanticipated recruitment challenges, however, the sample size was smaller, which is another study limitation. Recruitment strategies, therefore, needed to be tailored for each church based on church culture, internal systems, and leadership structure. Church leadership and potential study participants were nearly universally enthusiastic about the study, however recruitment required more steps than anticipated, such as development of several different forms of recruitment materials and presentation to and approval by the church’s governing body or health ministry. It therefore required more time than anticipated, a key learning for future studies.

Overall, study results suggest the cultural appropriateness and efficacy of civic engagement as an approach to behavior change of Black/African American women. Significant improvements in behavioral outcomes such as self-reported physical activity, cardiovascular fitness, and systolic blood pressure were realized. The small sample size suggests that the results should be interpreted with caution, however these noteworthy and promising results provide justification for a larger study. Interestingly, subjective measures of physical activity improved significantly, objective accelerometry-based physical activity data changed in the expected direction but was not significant. This could be due to an increased awareness in physical activity habits as a result of participating in the study. As shown in the significant decreases in their pre-post walk times, the study participants also improved their cardiorespiratory fitness. Overall, the study demonstrated positive impacts on dietary intakes, which counters the (albeit non-significant) pre-post increases in weight. One possible explanation supports the fit and fat concept, in which study participants became fitter throughout the intervention, despite not losing weight [62]. There was also an unexpected pre-post increase in waist circumference, which was statistically significant. A growing body of literature suggests an association between abdominal adiposity, obesity, and stress through the action of increased cortisol, which could provide a possible explanation for the observed results of increased perception of stress and waist circumference [63, 64]. Future larger scaled studies may explore this possibility given the prevalence of stress and stress-related diseases in this population.

A major consideration in the interpretation of the results of this pre-post feasibility study is the lack of a comparison group and the small sample size. The changes found therefore cannot be definitively attributed to the intervention itself, given other possible contributing factors. Furthermore, participants received both the Change Club civic engagement curriculum as well as nutrition education based on an effective heart health program for women [33]. However, only education was provided and behavioral components such as skill-building and actual physical activity were not included. It is therefore unlikely that the education alone was responsible for changes in outcomes. A larger, randomized, controlled trial with three study arms (control, nutrition education only, and the Change Club civic engagement intervention) would fully resolve this question.

## Conclusions

Overall, this novel behavior change intervention involving civic engagement shows promise as an effective, culturally-appropriate approach not only to implement individual-level changes of the study participants but also individuals of the broader community in order to address heart disease among Blacks/African Americans overall.

## Abbreviations

AACORN: African American Collaborative Obesity Research Network
IPAQ MET: International Physical Activity Questionnaire Metabolic Equivalent
PAR: Physical activity recall
